# The 3‐item short version of the Bayer‐ADL scale as a screening tool for distinguishing mild cognitive impairment from dementia in primary care

**DOI:** 10.1002/alz70857_107757

**Published:** 2025-12-26

**Authors:** Sophia Holzhauser, Oliver Goldhardt, Patrick Sommer, Thomas Wegehaupt, Ricarda Böhle, Lea Henrichs, Timo Grimmer

**Affiliations:** ^1^ Technical University of Munich, School of Medicine and Health, TUM University Hospital, Center for Cognitive Disorders, Munich, Bavaria, Germany

## Abstract

**Background:**

The increasing global prevalence of dementia, where early intervention can significantly impact treatment outcomes and costs, necessitates effective early diagnostic tools, particularly in primary care settings.

This study evaluates the Bayer Activities of Daily Living (Bayer‐ADL) scale as a potential screening tool to distinguish between Mild Cognitive Impairment (MCI) and dementia.

**Method:**

Conducted at the Center for Cognitive Disorders, Technical University of Munich, the study analyzed data from 503 patients who also had a comprehensive diagnostic work‐up including a standardized assessment of the presence and severity of dementia, using the Clinical Dementia Rating scale (CDR) and a thorough neuropsychological examination using the Consortium‐to‐Establish‐a‐Registry‐in‐Alzheimer’ Disease neuropsychological assessment battery (CERAD‐NAB) using bivariate correlations, linear regression, and ROC curves. To select key questions of the ADL scale a stepwise variable selection process was applied.

**Result:**

The findings demonstrate that three key questions of the Bayer‐ADL scale effectively differentiate MCI from dementia with high predictive accuracy (AUC = 0.836). The 3‐tem short version offers comparable predictive power to the full scale (37% each), suggesting its utility in primary care for streamlined dementia assessment.

**Conclusion:**

Consideration should be given to offering the 3‐item short version of the questionnaire to improve the efficiency and accuracy of dementia screening in primary care settings.

